# The potato mop-top virus TGB2 protein and viral RNA associate with chloroplasts and viral infection induces inclusions in the plastids

**DOI:** 10.3389/fpls.2012.00290

**Published:** 2012-12-24

**Authors:** Graham H. Cowan, Alison G. Roberts, Sean N. Chapman, Angelika Ziegler, Eugene I. Savenkov, Lesley Torrance

**Affiliations:** ^1^The James Hutton Institute, InvergowrieDundee, Scotland, UK; ^2^Federal Research Centre for Cultivated Plants, Julius Kühn Institute, Institute for Epidemiology and Pathogen DiagnosticsQuedlinburg, Germany; ^3^Department of Plant Biology and Forest Genetics, Swedish University of Agricultural SciencesUppsala, Sweden

**Keywords:** PMTV, TGB2, viral RNA, replication, chloroplasts, inclusions

## Abstract

The potato mop-top virus (PMTV) triple gene block 2 (TGB2) movement proteins fused to monomeric red fluorescent protein (mRFP-TGB2) was expressed under the control of the PMTV subgenomic promoter from a PMTV vector. The subcellular localizations and interactions of mRFP-TGB2 were investigated using confocal imaging [confocal laser-scanning microscope, (CLSM)] and biochemical analysis. The results revealed associations with membranes of the endoplasmic reticulum (ER), mobile granules, small round structures (1–2 μm in diameter), and chloroplasts. Expression of mRFP-TGB2 in epidermal cells enabled cell-to-cell movement of a TGB2 defective PMTV reporter clone, indicating that the mRFP-TGB2 fusion protein was functional and required for cell-to-cell movement. Protein-lipid interaction assays revealed an association between TGB2 and lipids present in chloroplasts, consistent with microscopical observations where the plastid envelope was labeled later in infection. To further investigate the association of PMTV infection with chloroplasts, ultrastructural studies of thin sections of PMTV-infected potato and *Nicotiana benthamiana* leaves by electron microscopy revealed abnormal chloroplasts with cytoplasmic inclusions and terminal projections. Viral coat protein (CP), genomic RNA and fluorescently-labeled TGB2 were detected in plastid preparations isolated from the infected leaves, and viral RNA was localized to chloroplasts in infected tissues. The results reveal a novel association of TGB2 and vRNA with chloroplasts, and suggest viral replication is associated with chloroplast membranes, and that TGB2 plays a novel role in targeting the virus to chloroplasts.

## Introduction

Plant viral genomes are relatively small and most comprise positive-sense, single-stranded RNAs that encode a few multi-functional proteins. RNA viruses replicate in association with various cellular membranes including those of the endoplasmic reticulum (ER), mitochondria, peroxisomes, vacuole, and chloroplasts (reviewed by Ahlquist et al., 2003; Salonen et al., 2005; Harries et al., 2010; Laliberté and Sanfaçon, 2010). Targeting of viral replication machinery to particular compartments by virus-encoded, non-structural proteins that associate with specific organellar membranes, can lead to the formation of membrane-bound, cytosolic viral replication complexes (VRCs). For those viruses, such as potato mop-top virus (PMTV), where the coat protein (CP) is not required for cell-to-cell or systemic movement (Torrance et al., 2009), different membranes may be involved in replication and movement of the viral ribonucleoprotein (vRNP) complex and virions. In addition, plant viruses encode one or more movement proteins to facilitate the spread of infection throughout the plant (reviewed by Lucas, 2006; Taliansky et al., 2008; Schoelz et al., 2011). It is becoming apparent that some movement proteins are multi-functional, playing additional roles in virus replication, counter defense and pathogenicity, as well as in viral genome transport (Scholthof, 2005; Lucas, 2006; Torrance et al., 2006).

PMTV, the type species of the genus *Pomovirus* is a tubular rod-shaped virus with a tripartite, positive-sense, single-stranded, RNA genome. The PMTV genome contains a module of three overlapping open reading frames (ORFs) encoding the triple gene block proteins (TGB), that are required for virus movement. The TGB module is found in nine genera of plant viruses and previously was classified into two broad groups: potex-like and hordei-like (reviewed by Morozov and Solovyev, 2003). However, research has shown that there are some differences in functional properties and this has led to a revision and re-classification into three groups: potex, hordei, and pomovirus (Verchot-Lubicz et al., 2010). The TGB of PMTV represents the Pomovirus group. In all groups, the three TGB movement proteins act in a coordinated manner, and all are required for cell-to-cell and systemic movement of the viral genomes. The hordei- and pomo-like TGB viruses are differentiated from the potex-like viruses by their larger TGB1 containing an N-terminal domain of variable mass in addition to the helicase domain, the TGB3 which contains two predicted transmembrane domains and the CP is not needed for systemic movement. Furthermore, there is increasing evidence that there are subtle differences in the sub-cellular localizations of the TGB proteins between the hordei-, pomo-, and potex-like groups which may indicate differences in functional roles (Haupt et al., 2005; Ju et al., 2005, 2007; Verchot-Lubicz, 2005; Torrance et al., 2006; Lim et al., 2009; Tilsner et al., 2010; Wright et al., 2010). In this paper we focus on the pomovirus PMTV TGB2. The current model for the coordinated action of the TGB proteins is that TGB1 interacts with viral RNA, forming a ribonucleoprotein vRNP movement complex that exits the cell, and that the TGB1/vRNP complex requires the integral membrane proteins TGB2 and TGB3 for localization to the plasma membrane and plasmodesmata (PD; Zamyatnin et al., 2004; Lucas, 2006; Lim et al., 2008; Verchot-Lubicz et al., 2010). Studies using TGB2 and TGB3 fused to fluorescent proteins and expressed from the cauliflower mosaic virus 35S (35S) promoter have revealed that they co-localize in cellular membranes and mobile granules and utilize the actin-ER network to facilitate movement to the cell periphery and PD (Gorshkova et al., 2003; Haupt et al., 2005). PMTV TGB2 and TGB3 contain vesicle-targeting and PD-targeting signals respectively, enabling them to associate with components of the endocytic pathway (Haupt et al., 2005; Tilsner et al., 2010). Although TGB2 and TGB3 can both increase the size exclusion limit (SEL) of PD, there is no evidence that they are independently capable of trafficking between cells (Haupt et al., 2005). The properties of PMTV TGB2 differ from those of hordeivirus TGB2 proteins reported to date in that it binds RNA in a sequence non-specific manner (Cowan et al., 2002) but is not required for RNA replication, can increase PD SEL independently (Haupt et al., 2005), and it associates with components of the endosomal network (Haupt et al., 2005).

There have been many reports of plastids as possible sites of virus replication. The turnip mosaic potyvirus (TuMV) has been shown to sequentially recruit the ER and chloroplasts for genome replication, and the 6K protein, which induces ER-derived vesicles, viral RNA and replicase components have all been found associated with invaginations of the plastid envelope (Jakubiec et al., 2004; Wei et al., 2010). Chloroplasts are also amalgamated into perinuclear bodies containing other organelles and viral proteins in TuMV-infected tissue (Grangeon et al., 2012). For turnip yellow mosaic virus (TYMV), virus particles, the replicase protein, CP and viral RNA have all been found associated with cytoplasmic inclusions that form at the periphery of chloroplasts and have been proposed to be the sites of RNA replication and viral encapsidation (Ushiyama and Matthews, 1970; Hatta et al., 1973; Hatta and Matthews, 1974, 1976; Prod'homme et al., 2001, 2003). Barley stripe mosaic virus (BSMV), closely related to PMTV, also causes invaginations in chloroplast membranes which contain virions (Carroll, 1970; McMullen et al., 1978) and viral proteins TGB2 and γb (Torrance et al., 2006), suggesting that plastids are possible sites of BSMV replication and virion assembly (Lin and Langenberg, 1984, 1985). The TGB3 protein of *Alternanthera* mosaic virus (AltMV) has also been shown to target to chloroplasts and the targeting is required for both efficient cell-to-cell and long-distance movement of that virus (Lim et al., 2010). Ultrastructural changes and production of peripheral vesicles in chloroplasts have also been found for a wide range of other plant viruses although direct evidence of viral replication or assembly has not always been detected.

To date, the published experimental data describe the localizations of PMTV TGB2 when expressed from the 35S promoter. This paper presents results of localization studies of proteins expressed from a viral subgenomic promoter in a virus context and establishes the functionality of the mRFP-TGB2 fusion protein in facilitating cell-to-cell movement. The confocal laser-scanning microscopy results are supported by biochemical and ultra-structural studies of PMTV-infected tissues which reveal that PMTV infection induces chloroplast abnormalities and that viral CP, RNA, and TGB2 are associated with chloroplasts. Collectively, the results allow us to hypothesize that chloroplasts may be sites of virus replication and possibly encapsidation, and that TGB2 may play a role in directing movement of both viral RNP complexes and virions.

## Materials and methods

### Plant material

*Nicotiana benthamiana* plants were grown from virus-free seed stocks maintained at The James Hutton Institute (JHI, UK) and sown in 10 cm diameter pots in a compost mix containing: 66% (v/v) Irish moss peat, 28% (v/v) Vermiculite, 6% (v/v) Pavoir sand, 0.14% (w/v) magnesium limestone, 0.14% (w/v) calcium limestone, 0.17% (w/v) Sincrocell controlled release fertilizer (William Sinclair Horticulture Ltd, Lincoln, UK), 0.08% (w/v) Osmocote Start fertilizer (Scotts, Humberside, UK) and 0.03% (w/v) Celcote wetting agent (LBS Horticulture, Lancashire, UK) and growth of plants was maintained in a glasshouse with a daytime temperature of 26°C and night time of 22°C. The daytime light intensity varied between 400 and 1000 μmol m^−2^ sec^−1^ with a mean day length of 16 h. Transgenic *N. benthamiana* plants expressing ER-targeted GFP (35S::mGFP5ER-HDEL) were a gift from Jim Haseloff (Haseloff et al., 1997) and grown under the same conditions as above.

### Transient expression plasmids

The 35S::mRFP-TGB2 vector used by Haupt et al. (2005) contained the gene encoding PMTV TGB2 fused to the 3′-end of the mRFP ORF and placed under control of the 35S promoter in the plasmid vector pRTL2. This plasmid was modified to prevent expression of the N-terminal portion of TGB3 by mutating the AUG initiation codon of the overlapping TGB3 ORF to ACG. A fusion of green fluorescent protein (GFP) to aspartate aminotransferase (GFP-AAT) was used as a marker for the plastid stroma (Kwok and Hanson, 2004).

### Infectious clones and transcript preparation

Full-length cDNA clones of the three genomic RNAs of a Swedish isolate of PMTV (PMTV_Sw_), named pPMTV-1, pPMTV-2, and pPMTV-3 (Savenkov et al., 2003) were used to produce run-off transcripts as follows: *Mlu*I-linearized (pPMTV-1 and pPMTV-2) or *Spe*I-linearized (pPMTV-3) plasmid DNA was used as template in the RiboMax Large Scale RNA Production System (Promega). The transcripts were mixed with an equal volume of GKP buffer (50 mM Glycine, 30 mM K_2_HPO_4_, 3% (w/v) celite, 3% (w/v) bentonite) and mechanically inoculated onto leaves of 4-week old *N. benthamiana* plants. Three weeks post inoculation the systemically-infected leaves were examined or used as sources of inoculum.

### Expression of GFP-TGB2 from a TMV vector

The recombinant TMV vector GFP-TGBp2 (Cowan et al., 2002) was used to infect 4-week old *N. benthamiana* plants. At 6 days post inoculation (dpi) systemically-fluorescing leaves were sampled and used for plastid preparation (see later).

### Plasmid construction to create PMTV reporter and mutant clones

The plasmid pPMTV-3 (Savenkov et al., 2003; see above) was used to generate the constructs described in this study. To demonstrate that a 400 nt fragment upstream of the TGB2 gene could function as a subgenomic promoter for expression of a reporter protein, the putative subgenomic promoter fragment and YFP gene were inserted downstream of the TGB3 gene to produce pPMTV3.sgP2::YFP. First *Nco*I and *Apa*I restriction enzyme sites were inserted downstream of the TGB3 gene through PCR. The resulting plasmid was digested with *Nco*I and *Apa*I and ligated to a *Nco*I/*Apa*I-digested YFP PCR product obtained using primers (1) (5′-GCCACCATGGTGAGCAAGGGCGAGGAGC-3′) and (2) (5′-AAAGGGCCCTTACTTGTACAGCTCGTCCA-3′) to yield pPMTV3.YFP. Finally, an *Nco*I-digested PCR product, encompassing the putative subgenomic promoter (1361–1745 nt in RNA3), obtained using primers (3) (5′-CTGCCAT*GGATCC*GATTTGGTAAAGCTACAGC-3′) and (4) (5′-GGACCATGGCTGTCTGTTTGTGGTTGC-3′), was cloned into *Nco*I-digested pPMTV3.YFP. The correct orientation of the putative subgenomic promoter was verified using a unique *Bam*HI restriction site (in italics, above). Inoculation of RNA transcripts onto *N. benthamiana* leaves resulted in detectable YFP fluorescence, demonstrating that the putative subgenomic promoter was functional. To generate an RNA-based replicon with enhanced stability, the plasmid pPMTV3.sgP2::YFP was digested with *Mlu*I and *Bam*HI (removing a portion of the TGB1 gene and all of the TGB2 and TGB3 genes) prior to blunting and self-ligation to yield the replicon plasmid pR3.TGB1(5′).sgP2::YFP.

To substitute a mRFP-TGB2 gene fusion for the YFP gene, a *Nco*I/*Apa*I-digested mRFP-TGB2 PCR product, generated with primers (5) (5′-CGACCATGGCCTCCTCCGAGGACGTC-3′) and (6) (5′-TTTGGGCCCTCTAGATTAACCTCCATATGAC-3′), was cloned into *Nco*I/*Apa*I-digested pR3.TGB1 (5′).sgP2::YFP to produce pR3.sgP2::mRFP-TGB2. Subsequently, the start codon of the TGB1 gene was removed to prevent expression of a truncated TGB1. The 5′-UTR was amplified with primers (7) (5′-AAAGGTACCTAACAAGGAATCGTGAAACAATT-3′) and M13-Reverse, and digested with *Sac*I/*Kpn*I prior to cloning into *Sac*I/*Apa*I-digested pR3.sgP2::mRFP-TGB2 with a *Kpn*I/*Apa*I-digested PCR product comprising the entire cassette (5′ end of the TGB1 gene, subgenomic promoter and mRFP-TGB2 gene fusion) obtained with primers (6) (see above) and (8) (5′-AAAGGTACCGAAAGCGCATTCAACGGAAG-3′). The resulting plasmid, pR3.ΔATG.sgP2::mRFP-TGB2, was used in mRFP-TGB2 localization studies.

To construct pPMTV-3.YFP-TGB1, the plasmid pPMTV-3.GFP-TGBp1 (Zamyatnin et al., 2004) was digested with *Nco*I to remove the GFP gene and ligated to a *Nco*I-digested YFP PCR product obtained using primers (1) (see above) and (9) (5′-AAACCAT*GGATCC*CTTGTACAGCTCGTCCATG-3′). The correct orientation of the YFP gene was verified using a unique *Bam*HI restriction site (in italic, above).

Primers PMTV13MutF (5′-CCGGCCAATATTAATTTGGTCGCGC-3′) and PMTV13MutR (5′-GCGCGACCAAATTAATATTGGCCGG-3′) were used to prepare the construct pPMTV-3.YFP-TGB1.ΔTGB2, in which the A at nucleotide 37 in the TGB2 coding sequence was converted to a T. This created an in-frame UAA stop codon and prevented expression of TGB2 without interfering with expression of TGB1 or TGB3.

### Microprojectile bombardment

Plasmid DNA and RNA transcripts were introduced into leaf epidermal cells by bombardment of gold particles as described previously by Haupt et al. (2005).

### Agrobacterium-based transient expression

The binary vector pGreen0229 (Hellens et al., 2000) was modified by insertion of the T-DNA cassette from pRTL2 to produce pGRAB (Petra Boevink, unpublished). The mRFP-TGB2 gene fusion was cloned into pGRAB for transformation of *Agrobacterium tumefaciens* strain LBA4404. Bacterial cultures were grown and, after testing across a wide range of optical densities (*OD*_600_ = 0.01–0.5) all of which gave the same phenotype, the cells were suspended at *OD*_600_ = 0.2 in 10 mM MES (pH 5.5), 10 mM MgCl_2_ containing 0.15 mM acetosyringone for 1 h at room temperature (~22°C) before infiltration through stomata on the abaxial surface of *N. benthamiana* source leaves.

### Confocal laser-scanning microscopy

Confocal images were obtained using a Leica TCS SP2 spectral confocal laser-scanning microscope (CLSM) equipped with water-dipping lenses. GFP and YFP were excited at 488 nm, and emission collected between 505–530 and 515–535 nm, respectively. mRFP was excited at 561 nm and emission collected between 580–600 nm. Chlorophyll autofluorescence was excited at 488 nm and emission collected between 660–700 nm. Simultaneous imaging of GFP and mRFP fusions, or YFP and mRFP-fusions was possible, but imaging of chlorophyll in combination with mRFP was always performed sequentially.

### CLSM image deconvolution

For deconvolution, images were collected using an active Z-galvo attachment for the Leica SP2 CLSM, 512 × 512 pixel resolutions and a voxel size of approximately 75 nm to suit the XY resolution of the Leica HCX PL APO 63x water-dipping lens. Images were recorded using a Z-step size equivalent to 0.5–1 times the z-resolution of the objective lens. Point spread functions (PSF) were calculated for each fluorophore by imaging fluorescent beads with appropriate fluorescent characteristics (PS-Speck microscope point source kit; Molecular Probes) and processing the data using Amira 3D visualization software (Mercury Computer Systems Inc.). Deconvolution of the images was also carried out using Amira software, utilizing the appropriate PSFs. Multiple-channel, 3D deconvolved images were separated into single channels and transferred to Adobe Photoshop (Adobe Corporation) software to create the 2D projections shown in Figure 3.

### Plastid preparations

Four week old *N. benthamiana* plants were inoculated with PMTV_Sw_ and maintained in a growth cabinet providing a 14 h, 18°C light period with light intensity of 145 μmol m^−2^ s^−1^, and a 10 h, 15°C dark period. Plastids were isolated essentially as described by Nivison et al. (1986) from plants 14–20 dpi following a 24 h dark period. Briefly, 1 g of leaves was triturated in seven volumes of grinding buffer (0.35 M Sorbitol, 0.05 M HEPES-KOH pH 7.5, 2 mM EDTA, 0.5 mM MgCl_2_, 1 mM DTT, 10 mg/mL BSA) and filtered through muslin. The extract was centrifuged at 1000× g for 3 min (Eppendorf centrifuge 5417R; rotor F45-30-11) and the resulting pellets suspended in a total of 1 ml Sorbitol medium (0.35 M Sorbitol, 35 mM HEPES-KOH pH 8.3, 10 mM K_2_HPO4, 0.5 mM MgCl_2_, 1 mM DTT). A 0.5 ml aliquot of the preparation was layered onto a gradient comprising 1 ml of 40% and 1 ml 85% [v/v] Percoll (prepared in 40 mM HEPES-KOH pH 7.5, 0.05 mM MgCl_2_, 0.35 M Sorbitol, 1 mM DTT) and centrifuged at 13,000× g for 7 min. The intact plastids were recovered from the interface between the 40% and 85% layers of Percoll, and a fraction containing broken chloroplasts was recovered from a single band in the 40% Percoll fraction. The plastid samples were washed twice by dilution with five volumes of Sorbitol medium and centrifugation at 4000× g for 5 min. The pellet containing the plastids was finally suspended in 0.5 ml Sorbitol medium.

### Western blots

Samples of leaf extracts and plastid preparations were mixed with an equal volume of Laemmli buffer and electrophoresed in a 12.5% SDS-PAGE gel. The separated proteins were electroblotted onto Hybond ECL nitrocellulose (GE Healthcare) and the membrane subsequently incubated with antibody preparations essentially as described previously (Torrance, 1992) using the immunoglobulin fraction of a PMTV antiserum (Torrance et al., 1993) at 2 μg ml^−1^ and an anti-rabbit alkaline phosphatase conjugate (Sigma product no A8025) as the second antibody.

### RT-PCR

Total RNA was extracted from leaf or plastid samples using the RNeasy Plant Mini Kit (Qiagen) following the manufacturer's instructions. First strand PMTV RNA1 cDNA was synthesized with the reverse primer (5′-CGATCGTGTCTTGATCGCAGC-3′) using one microgram of total RNA. The resulting cDNA was used as template (2 μl of a 1:10 and 1:100 dilution in a 25 μl reaction) in a PCR with the reverse primer (above) and forward primer 5′-CTTGTGGGAGAAGTCGCAGTG-3′ to amplify a product of 1057 bp.

### Detection of negative-strand RNA associated with chloroplasts

Chloroplasts were isolated from PMTV-Swe-infected and non-infected *N. benthamiana* leaves and counted using a haemocytometer. Aliquots containing 4, 2, 1, 0.5, or 0.25 × 10^7^ chloroplasts were taken and total RNA extracted using the RNeasy Kit (Qiagen) then 10 μl used as template to prepare cDNA using M-MLV reverse transcriptase (Promega) with 10 pmol of primer Nested1 (5′-GTGAATGCGATACTTCACAC-3′) in 20 μl reaction. 2 μl of cDNA was used as template in a 50 μl PCR reaction comprising 1× Green GoTaq Reaction buffer (Promega), 0.2 μM of primer Nested2 (5′CACTTACGCTATGAAGTGTG-3′) and 0.2 μM of primer Nested3 (5′-GTCACATACAACATCAACGAG-3′), 0.2 mM dNTPs, 2.5 mM MgCl_2_, and 2.5 μl of GoTaq DNA Polymerase (Promega). The PCR conditions were 95°C for 2 min, then 30 cycles of 95°C for 30 s, 55°C for 30 s, 70°C for 30 s, followed by 70°C for 10 min. 2 μl of this reaction were used as template for a second PCR with primers Nested6 (5′-GACATCTTCAGTGCACAGAGG-3′) and Nested7 (5′-GTAAAACCCATTGACGCTAGG-3′) using the conditions 95°C for 2 min, then 35 cycles of 95°C for 30 s, 55°C for 30 s, 70°C for 30 s, followed by 70°C for 10 min. These PCR products were electrophoresed in 2% agarose gels and then stained with ethidium bromide.

### *In-situ* RNA hybridisation

RNA probes were created by cloning a 950 bp fragment from the 3′ UTR of PMTV RNA1 into the pGEMT-Easy vector. PCR screening was used to identify clones containing the sequence in both orientations such that both sense and antisense RNA probes could be produced by running off transcripts from the T7 promoter, ensuring comparable quantity and quality of both probes. The *in-situ* hybridization protocol was a modified version of that used by Drea et al. (2005), brief details of which follow.

Tissue was fixed in 4% paraformaldehyde in PEM buffer (0.1 M PIPES pH 6.95, 1 mM EGTA, 1 mM MgSO_4_), dehydrated through an ethanol series and then into xylene before being infiltrated with and then embedded in Paramat Extra wax (VWR International, Lutterworth, UK). Chloroplasts were prepared as previously described and embedded in 1% Agar No.1 (Oxoid Ltd., Basingstoke, UK) before being fixed and processed in parallel to the plant tissue. Tissue and chloroplasts were sectioned to 10 μm thick and mounted on Polysine slides (Menzel–Glaser, Braunschweig, Germany) before being de-waxed and pre-treated as follows: two washes in PBS buffer, two washes in Histoclear (Fisher Scientific, Loughborough, UK), for 20 min each; 100% ethanol for 10 min, then through a 95, 85, 50, and 30%, ethanol series (2 min each); PBS for 3–4 min; proteinase K treatment (2–3 μg/mL in 100 mM Tris and 10 mM EDTA, pH 7.5) for 30 min at 37°C; Glycine (0.2%, w/v) in PBS for 2 min; PBS for 3–4 min; acetic anhydride (0.5% [v/v] in 0.1 M triethanolamine, pH 8.0) for 10 min; PBS for 3–4 min; and then back through 30, 50, 85, 95, and 100% ethanol. Slides were dried at room temperature and stored at 4°C until hybridization.

*In vitro* transcription was performed for 2 h at 37°C incorporating digoxigenin-UTP nucleotides (0.35 mM), using T7 RNA polymerase (Promega) in the presence of 100 mM DTT, RNAsin and 200 ng of PCR product as the DNA template. (The plasmid templates were cut using restriction enzyme *Pvu*II and then amplified using M13 forward and reverse primers). RNA probes were hydrolyzed immediately in 100 mM carbonate buffer, (60 mM Na_2_CO_3_, 40 mM NaHCO_3_, pH 10.2), at 60°C for 30 min to reduce the probe to ~200 bp fragments, and products were precipitated in 2.5 M ammonium acetate and three volumes of 100% ethanol for 1 h at 4°C. Following precipitation, transcripts were diluted in four times the volume of the original transcription reaction in RNAse-free water. To assess the incorporation of digoxigenin-UTP, probes were diluted and one microliter was spotted on nitrocellulose for a dot blot and processed as follows: 30 min in blocking buffer (Sigma–Aldrich, St. Louis, MO); 30 min in anti-digoxigenin–alkaline phosphatase-conjugated antibody (Roche, Herts, UK; diluted 1/5000 in TBS); 5-min wash in Tris-buffered saline (TBS; 10 mM Tris and 250 mM NaCl); 5 min in alkaline phosphatase buffer (100 mM Tris, 100 mM NaCl, pH 9.5, and 50 mM MgCl_2_); and developed in alkaline phosphatase buffer containing nitroblue tetrazolium (0.1 mg/mL) and 5-bromo-4-chloro-3-indolyl phosphate salt (0.075 mg/mL) for ~10 min.

Hybridization chambers (Grace Biolabs) were applied securely to the slides (after pretreatment). The probes were diluted 100 times in hybridization solution (300 mM NaCl, 10 mM Tris, pH 6.8, 10 mM NaPO_4_, 5 mM EDTA, 50% [v/v] formamide, 5% [w/v] dextran sulfate, 0.5 mg/mL tRNA, 1× Denhardt's solution) and introduced into the chambers at 55°C after being heated to 85°C for 2 min to denature the probe. Hybridization was performed overnight in a 55°C incubator.

Chambers were removed and the slides were washed as follows: three 1 h washes in 0.2 × SSC (1 × SSC is 0.15 M NaCl and 0.015 M sodium citrate) at 50°C with constant agitation; 10 min in 1 × TBS at room temperature. The slides were then transferred into racks for anti-digoxigenin staining as follows: 1% blocking solution (Roche) in TBS for 1 h; 1 × TBS containing a 1:2500 dilution of anti-digoxigenin–alkaline phosphatase and 0.025% [v/v] Tween-20 for 2 h; four 10 min washes in 1 × TBS; and 5 min in alkaline phosphatase buffer (0.1 M Tris, 0.1 M NaCl, and 50 mM MgCl_2_, pH 9.5). Then, the color reaction was developed in alkaline phosphatase buffer containing nitroblue tetrazolium (0.1 mg/mL) and 5-bromo-4-chloro-3-indolyl phosphate-*p*-toluidine salt (0.075 mg/mL) for up to 6 h. Slides were then washed several times in water to stop the reaction before being dried.

If necessary, tissue was lightly counterstained with 0.25% Alcian Blue 8GX (Sigma–Aldrich, Dorset, UK) in 3% acetic acid to show cellular structure before being mounted in DPX mountant (Sigma–Aldrich, Dorset, UK) under glass coverslips. Representative sections for each probe were photographed with a Leica DC500 digital camera on a Nikon Optiphot microscope under bright-field conditions.

### Protein-lipid overlay blots

Protein lipid overlay assays were done using Membrane Lipid Strips™ (Echelon Biosciences Inc.) following the manufacturer's instructions. Briefly, blocked membranes were incubated overnight with up to 20 μg/mL preparations of either thioredoxin-TGB2 or thioredoxin (Cowan et al., 2002). The membranes were then incubated for 1 h with anti-thioredoxin-TGB2 rabbit polyclonal antiserum (Cowan et al., 2002) diluted to 1 μ g/mL, followed by anti-rabbit horseradish peroxidase (HRP) conjugate (Invitrogen Ltd.) diluted 1:1000 and visualized with HRP ECL™ substrate (GE Healthcare) according to the manufacturer's instructions.

### Transmission electron microscopy (EM)

Source material was obtained from PMTV-infected *N. benthamiana* plants with yellowing symptoms that had been maintained in the controlled environment conditions described above. Small segments of leaves were fixed, dehydrated and embedded in Araldite resin (Agar Scientific) as described by Taylor et al. (2000). In addition, leaves were taken from naturally infected potato, *cv* Scarborough, showing aucuba (yellow mosaic) symptoms which had tested positive for PMTV in ELISA and EM examination failed to detect the presence of any other virus. Segments of potato leaf were fixed and dehydrated as described in Oparka et al. (1999), and embedded in LR White resin (Agar Scientific). Ultra-thin sections of both *N. benthamiana* and potato were mounted on pyroxylin-coated nickel grids, post-stained with uranyl acetate and lead citrate as described by Roberts (2002), and examined in a Jeol 1200EX electron microscope.

## Results

### Expression of mRFP-TGB2 in epidermal cells from a modified virus vector

**Figure 1:**
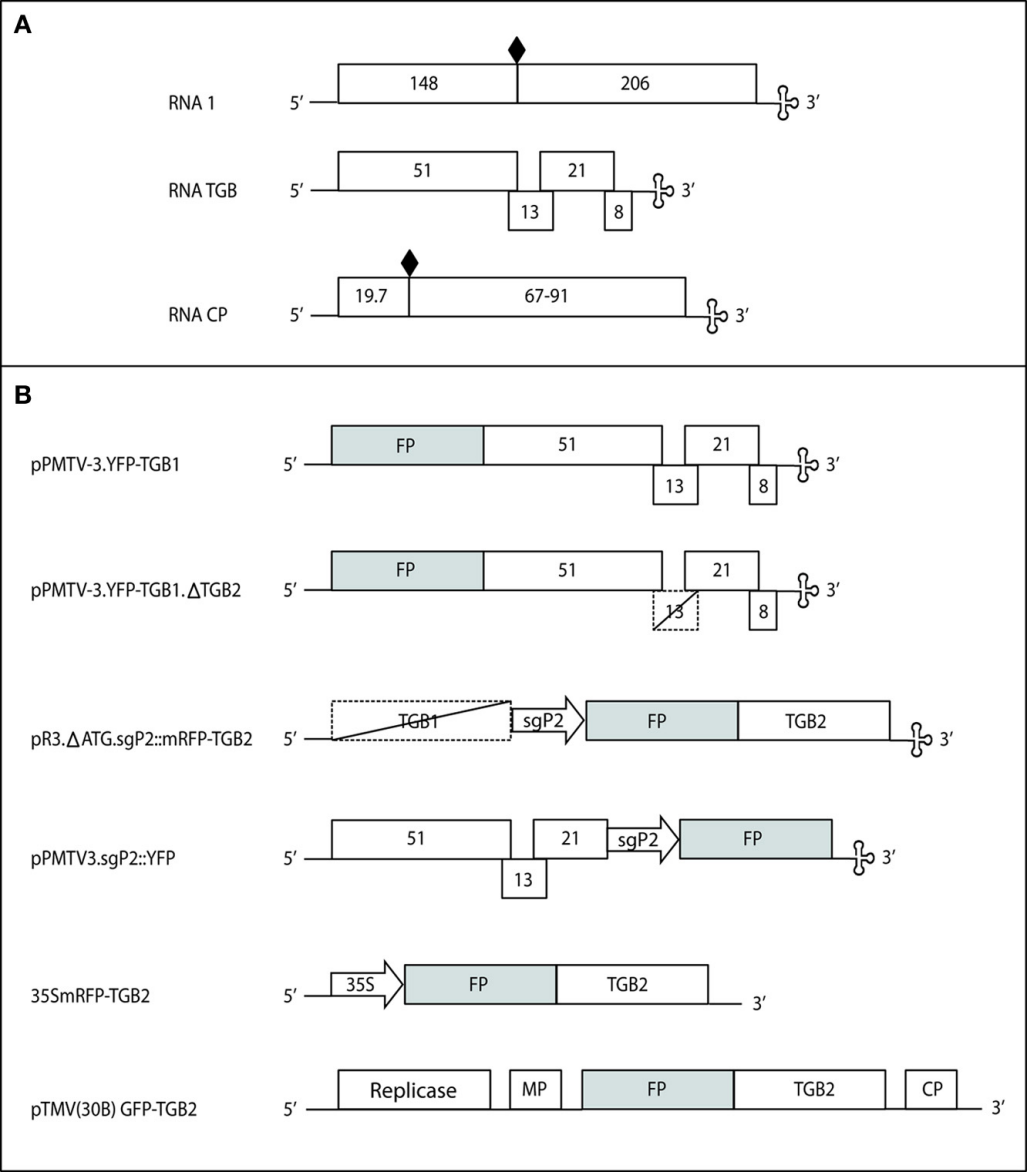
**PMTV genome (A) and constructs derived from RNA TGB (B).** The viral RNA1 encodes the 148K replication protein and the 206K read-through (RT) product. RNA TGB encodes the three overlapping TGB proteins (51K, 13K, and 21K) and an 8K cysteine-rich protein. RNA CP encodes the 19.7K coat protein and its RT domain. Diamonds, leaky termination codons; clover-leaf motif, tRNA-like structure; solid boxes, expressed ORFs, dashed boxes, non-expressed ORFs, promoters are shown in arrows, FP, fluorescent protein. The 35S mRFP-TGB2 diagram shows the gene fusion expressed from the plasmid pRTL2 (used for microprojectile bombardment) or the binary vector pGRAB (used for *Agrobacterium*-mediated transient expression).

**Figure 2:**
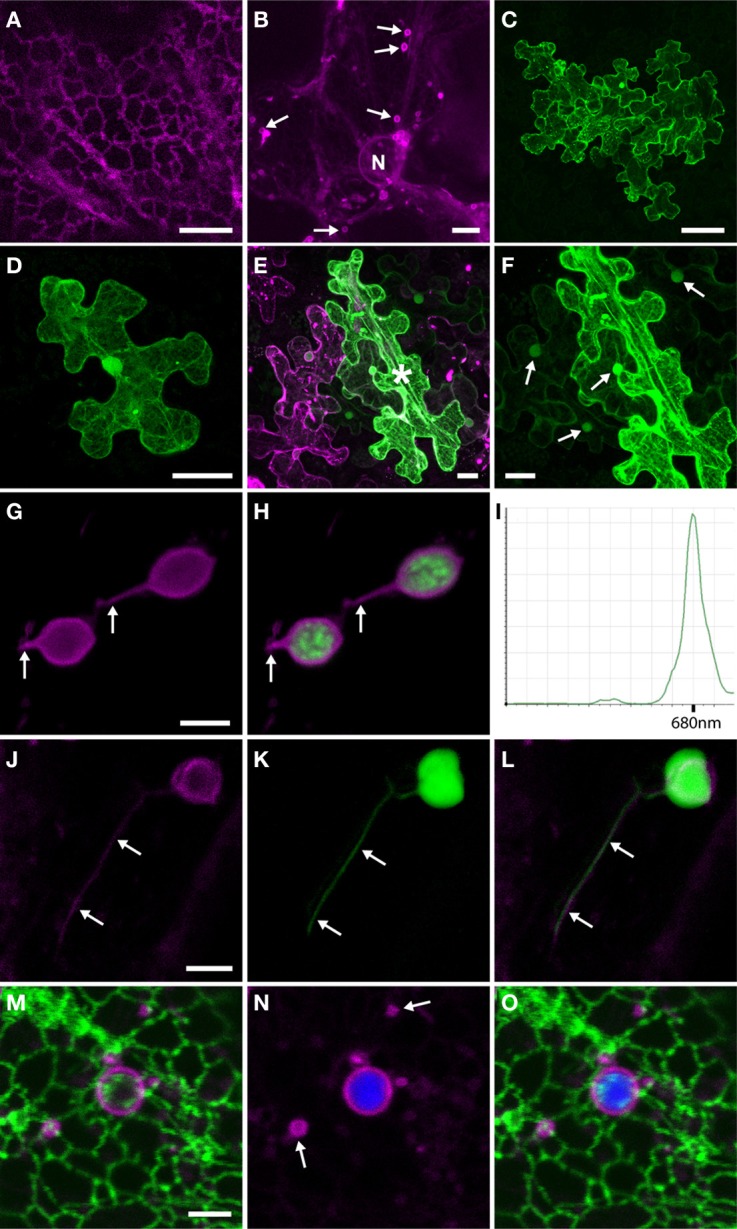
**Transient expression of TGB2 protein in epidermal cells. (A)** Expression of sg::mRFP-TGB2 (magenta) from a PMTV vector in epidermal cells of *N. benthamiana*. Fluorescence is first visible throughout the ER network (typically 1 dpi). Bar = 10 μm. **(B)** Slightly later in the infection process (typically 2 dpi) than seen in **(A)**, mRFP-TGB2 labels the membranes of small mobile vesicles that are 2 μm in diameter (arrows). Bar = 10 μm. **(C)** Expression of PMTV reporter clone PMTV3.YFP-TGB1 in epidermal cells of *N. benthamiana* at 2 dpi; YFP fluorescence (green) is seen throughout a small lesion consisting of ~12 cells. Bar = 50 μm. **(D)** Expression of PMTV reporter clone PMTV3.YFP-TGB1ΔTGB2 in epidermal cells of *N. benthamiana*. In the absence of a functional TGB2, the viral reporter clone (YFP fluorescence) is confined to a single cell. Bar = 20 μm. **(E)** Expression of PMTV reporter clone PMTV3.YFP-TGB1ΔTGB2 (green) in epidermal cells of *N. benthamiana* that are expressing 35S::mRFP-TGB2 (magenta); YFP fluorescence has spread out of the initial cell (marked with ^*^) to surrounding cells; YFP can be seen in the cytosol and accumulated in the nuclei of neighboring cells. Bar = 10 μm. **(F)** Enlarged portion of the YFP signal shown in **(E)** to show the movement of the fluorescence out of the initial cell, and the accumulation of this YFP in nuclei of neighboring cells (arrowed). Bar = 10 μm. **(G,H)** Expression of sg::mRFP-TGB2 from PMTV vector in epidermal cells of *N. benthamiana*. Red fluorescence (magenta) is localized to the chloroplast envelope. Stromules are arrowed. Chlorophyll autofluorescence is shown in green in **(H)**. Bar in G = 4 μm for **(G** and **H)**. **(I)** The emission spectrum of the contents of a 4 μm diameter vesicle labeled with mRFP-TGB2 shows a peak at 680 nm (emission range shown is from 495 to 755 nm). **(J,K,L)** Co-expression of 35S::mRFP-TGB2 (magenta; **J**) with GFP-AAT (green; **K**) in a chloroplast in an epidermal cell of *N. benthamiana*. TGB2 is present in the membrane of the chloroplast and the stromule **(J)**, while GFP is visible throughout the stroma of both plastid and stromule **(K)**. The stromule is arrowed in each image and **(L)** shows an overlay of the two channels. Bar in J = 4 μm for **(J–L)**. **(M,N,O)** To ensure that the ER membrane was not contributing to the fluorescence seen on the chloroplast membrane, 35S::mRFP-TGB2 (magenta) was expressed in mGFP5-ER (green) transgenic *N. benthamiana*. The green ER does not surround the chloroplasts and the plastids sit internal to the cortical ER network. Strands of ER can be seen to cross the surface of the chloroplasts **(M** and **O)**. The membranes of both 2 μm diameter vesicles (arrows) and a 4 μm diameter chloroplast are labeled with mRFP-TGB2 **(M–O)**, with the chlorophyll autofluorescence from the interior of the plastid shown in blue **(N** and **O)**. Bar in M = 4 μm for **(M–O)**.

Previously, we studied the localization of mRFP-TGB2 expressed in epidermal cells from the 35S promoter following biolistic bombardment of plasmid DNA (Haupt et al., 2005) and we have confirmed these observed localizations through *Agrobacterium*-mediated expression (unpublished results). To investigate whether the localizations we observed were influenced by over-expression from the 35S promoter, the mRFP-TGB2 fusion protein was expressed from a PMTV vector (see Figure 1 for a schematic of all constructs used in this study). A region of the PMTV TGB genetic module containing the putative subgenomic promoter for TGB2 and TGB3 was identified. A cDNA clone was modified to delete the ORFs and a mRFP-TGB2 fusion gene inserted downstream of the putative subgenomic promoter sequence. RNA transcripts from this construct were bombarded with RNA1 onto *N. benthamiana* leaves. In these experiments, the virally-expressed fusion protein was observed in epidermal cells, indicating that the promoter was functional. The distribution of the virally-expressed, red fluorescent TGB2-fusion protein was the same as when expressed from the 35S promoter revealing associations at first with membranes of the ER (Figure 2A) and mobile granules and later, the membranes of two populations of small (Figure 2B) and larger, ~4 μm diameter, vesicular structures as reported previously (Haupt et al., 2005).

### mRFP-TGB2 complements virus movement

We previously showed that the mRFP-TGB2 fusion protein could function to increase the SEL of PD (Haupt et al., 2005). To test whether the mRFP-TGB2 could also complement virus movement, a PMTV reporter construct that expressed yellow fluorescent protein (YFP) fused to the N-terminus of TGB1 (PMTV-3.YFP-TGB1) was prepared. When transcripts of this construct were inoculated together with RNA1, small, spreading, fluorescent lesions, ~12 cells in size, were visible at 2 dpi (Figure 2C) and had spread to more than a hundred cells by 7 dpi. A derivative construct, PMTV-3.YFP-TGB1.ΔTGB2, was prepared in which a stop codon was introduced to prevent translation of TGB2. Inoculation of RNA transcripts from this derivative with RNA1 resulted in single fluorescent cells (Figure 2D), movement out of the initial cell never occurred, confirming that TGB2 is required for movement but not for replication. To test the functionality of the mRFP-TGB2 fusion protein, *N. benthamiana* leaves were infiltrated with *Agrobacterium* containing the binary vector with the 35S::mRFP-TGB2 cassette prior to bombardment of RNA transcripts of PMTV-3.YFP-TGB1.ΔTGB2 into the infiltrated area. Cells expressing both fusion proteins were rarely detected; a total of 27 cells in three experiments. Most of these cells showed aggregated (and probably non-functional) mRFP-TGB2 protein, but in the nine cells without aggregation the YFP reporter clone moved out of the initial cell (9/27 cells), often showing a halo surrounding the initial cell and occasionally moving over several cell boundaries (Figures 2E,F). These experiments indicate that mRFP-TGB2 can functionally complement for the TGB2 deletion in the mutated clone PMTV-3.YFP-TGB1.ΔTGB2.

### mRFP-TGB2 localises to the chloroplast envelope

CLSM imaging revealed that the mRFP-TGB2, expressed from either the binary vector or a viral replicon, was localized in the bounding membrane of two populations of vesicles; one approximately 1–2 μm in diameter and a second population of ~4 μm diameter vesicle-like compartments (Haupt et al., 2005). In the present study we investigated the 4 μm diameter structures in more detail. The vesicles were excited using 488 nm light and scanned over a wavelength range of 495–755 nm. We found mRFP fluorescence located in the membrane (Figures 2G,H) and the emission spectrum of the contents was found to match that of chlorophyll A (maximum at 680 nm; Figure 2I). Furthermore, when mRFP-TGB2 was co-expressed with GFP-aspartate aminotransferase (GFP-AAT; a marker for the plastid stroma), mRFP-TGB2 was seen to surround GFP fluorescence that was within the compartment. mRFP-TGB2 was also associated with GFP in stromules (Figures 2J–L), indicating that mRFP-TGB2 associates with membranes of the chloroplast envelope. It was noted that in the experiments where mRFP-TGB2 was expressed from the viral subgenomic promoter, the localization to chloroplasts occurred later than when expressed from the 35S promoter; typically 1–2 dpi from the 35 s promoter but 3–4 dpi from the viral promoter.

To eliminate the possibility that the mRFP-TGB2 fluorescence was in the ER surrounding the chloroplast, rather than in the chloroplast envelope, 35S::mRFP-TGB2 was expressed in transgenic *N. benthamiana* plants expressing ER-localized GFP (mGFP5-ER-HDEL; Haseloff et al., 1997). In these experiments, GFP was clearly associated with the ER network and, though in close proximity to the mRFP-TGB2-labeled vesicles, did not co-localize with the vesicle membrane (Figures 2M–O). The different fluorescent signals were also clearly distinguished when viewed over time (supplementary Figure 1; Movie).

**Figure 3:**
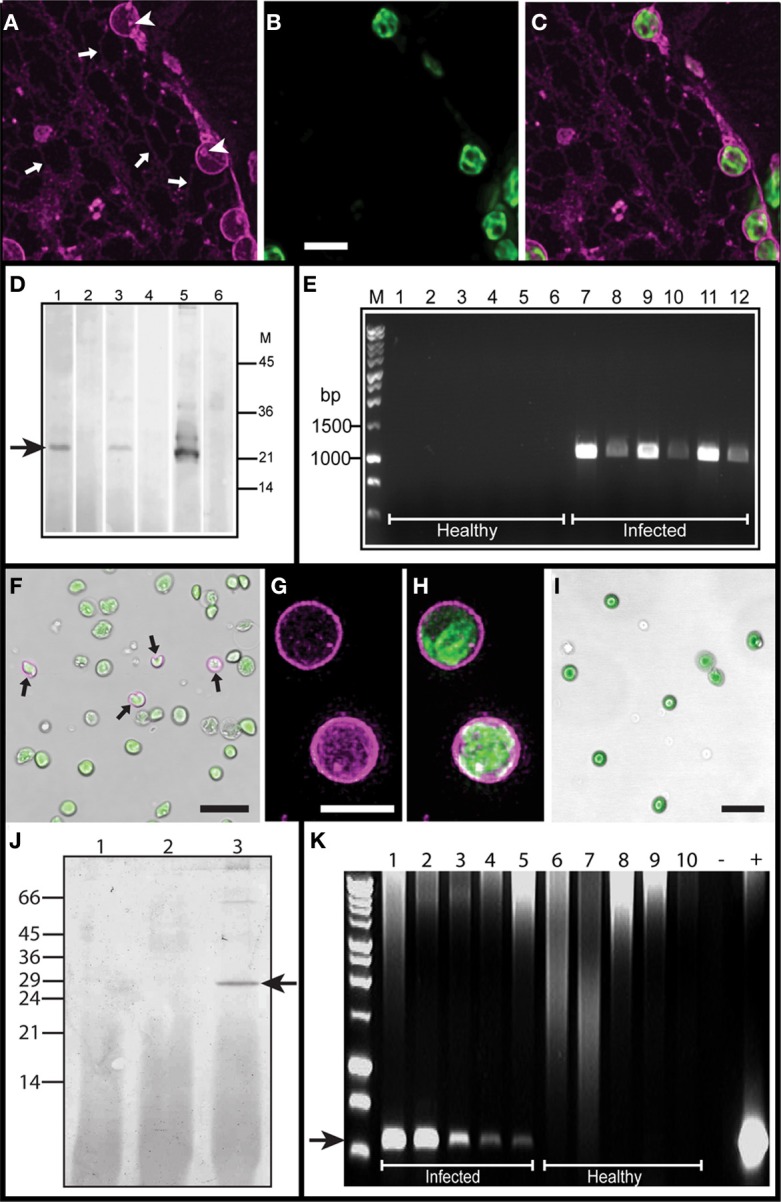
**Analysis of TGB2 protein associated with chloroplasts. (A,B,C)** Deconvolved images showing mRFP-TGB2 (in magenta; **A**) in the membrane of chloroplasts, in strands of the ER network (arrows) and in small bodies on the ER. Chlorophyll autofluorescence is shown in green **(B)**. TGB2 is also present at a low level in the stroma of the chloroplasts and in some foci on the surface of the plastids (arrowheads; **A**). **(C)** shows an overlay of **(A)** and **(B)**. Bar in B = 5 μm for **(A–C)**. **(D)** Western blot of plastid preparations from PMTV-infected (lanes 1, 3, 5) and non-infected (lanes 2, 4, 6) *N. benthamiana* leaves; the blot was reacted with anti-PMTV CP serum. Lanes 1 and 2, disrupted plastids and membrane fraction; lanes 3 and 4, intact plastids and lanes 5 and 6, non-fractionated preparation. Reaction with CP arrowed; the positions of the size markers (kDa) indicated on right. **(E)** RT-PCR of plastid preparations from non-infected (lanes 1–6) and PMTV-infected (lanes 7–12) *N. benthamiana* leaves. Total RNA was prepared from leaves (lanes 1, 2, 7, 8), intact plastid fraction (lanes 3, 4, 9, 10) or disrupted plastid and membrane fraction (lanes 5, 6, 11, 12). cDNA preparations from each sample were tested at dilutions of 1/10 and 1/100. Size markers indicated on the left. **(F)** False transmission and fluorescence image overlay of plastid preparation from *N. benthamiana* leaves infected with the TMV vector expressing GFP-TGB2. Chlorophyll autofluorescence is shown in green, while GFP-TGB2 on the plastid membrane is shown in magenta. ~5% of all plastids showed TGB2 in the outer membrane (arrows). Bar = 15 μm. **(G,H)** Higher magnification and deconvolved images of purified plastids labeled with GFP-TGB2 expressed from the TMV vector. The TGB2 signal is visible on the membrane and in small internal speckles (magenta; **G** and **H**) while the chlorophyll signal (green; **H**) is located in the thylakoids. Bar in G = 5 μm for **(G** and **H)**. **(I)** False transmission and fluorescence image overlay of plastid preparation from mGFP5-ER-HDEL *N. benthamiana* leaves. Chlorophyll autofluorescence is shown in green. No GFP fluorescence was detected in association with the plastid envelope. Bar = 15 μm. **(J)** Detection of ER proteins retained in chloroplast preparations from transgenic *N.benthamiana* expressing mGFP5-ER-HDEL. Western blot of disrupted plastid and membrane fraction (lane 1), intact chloroplasts (lane 2) and whole leaf extract (lane 3) reacted with anti-HDEL monoclonal antibody. The positions of molecular mass markers (kDa) are indicated to the left of the blot. **(K)** Detection of negative strand PMTV RNA1 in chloroplasts. Lanes 1–5 represent products amplified in RT-PCR from templates derived from PMTV-infected chloroplasts (4,2,1,0.5 and 0.25 × 10^7^ chloroplasts, respectively). Lanes 6–10 are controls showing no amplification from healthy chloroplasts (4,2,1,0.5 and 0.25 × 10^7^ chloroplasts, respectively). “−” represents a no template PCR control; “+” represents a positive control PCR reaction. The size of the arrowed DNA molecular mass marker (M) is 500 bp.

To clarify further the relationship of TGB2 with chloroplasts, 3-dimensional image stacks were acquired using microscope settings that allow deconvolution, giving improved image resolution. Following deconvolution, the structures of the chlorophyll-containing vesicles were more clearly resolved; the internal autofluorescence was located in thylakoids and grana stacks, confirming these structures were chloroplasts (Figures 3A–C). Furthermore, it was clear from the deconvolved image stacks that, although the mRFP-TGB2 labeling was most intense on the peripheral membrane of the chloroplasts, there was some signal from within the plastid (Figures 3A,C), indicating that some TGB2 was also present inside chloroplasts. Deconvolution of these images also showed that the ER network is still faintly labeled with mRFP-TGB2 (arrows in Figure 3A) when the protein begins to accumulate in plastids; something that was not visible previously, and similarly, small bright spots could be distinguished on the ER network in the deconvolved image. These latter objects are assumed to be the mobile granules typically found early in infection (see Haupt et al., 2005), as they are the same size, but, like the ER network, have reduced in fluorescence intensity to a level undetectable without deconvolution at the later time point when TGB2 protein accumulates in chloroplasts.

### PMTV RNA, CP, and TGB2 were detected in plastid preparations from *N. benthamiana* leaves

To investigate whether the chloroplast association occurred in the context of a natural viral infection, plastid preparations were made from leaves of uninfected and virus-infected *N. benthamiana* plants. Two fractions were obtained from Percoll step gradients; intact chloroplasts and a fraction containing membranes from disrupted plastids and other cellular membranes (membrane fraction). Western blots of the preparations revealed the presence of PMTV CP in samples of both the intact plastids (Figure 3D lane 3) and membrane fractions (also containing disrupted plastid membranes; Figure 3D lane 1); the band relating to the CP is arrowed. However, TGB2 could not be detected in these samples using antiserum prepared against TGB2 (data not shown).

Total RNA was prepared from each of the fractions and RT-PCR revealed the presence of viral genomic RNA in both intact plastids (Figure 3E, lanes 9 and 10) and membrane fractions (Figure 3E, lanes 11 and 12) as well as in total leaf extracts (Figure 2E, lanes 7 and 8). No viral RNA or CP was detected in negative control preparations from non-infected leaves (Figure 3E, lanes 1–6).

Chloroplasts were isolated from systemically-infected leaves of *N. benthamiana* infected with the tobacco mosaic virus (TMV) vector expressing GFP-TGB2 (Cowan et al., 2002). Western blot analysis of extracts from the isolated chloroplasts with the TGB2-specific antiserum failed to detect TGB2 accumulation. However, CLSM imaging of the intact isolated chloroplasts revealed that ~5% of chloroplasts were labeled with GFP-TGB2 (Figure 3F) although this is likely to be an underestimation because large starch grains in infected plastids cause them to rupture during chloroplast preparation, making it difficult to isolate infected plastids intact. Deconvolved image stacks of these isolated chloroplasts reconfirmed that the fluorescent TGB2 fusion protein was associated with the bounding membranes of the plastids (Figure 3G). Furthermore, these higher resolution images revealed that a small amount of TGB2-associated fluorescence was also present inside the plastids, as was chlorophyll auto-fluorescence (Figures 3G,H). In combination, these results suggest that the available TGB2 antiserum is not sensitive enough to detect the chloroplast-associated TGB2. In control experiments, plastid preparations from PMTV-infected mGFP5-ER-HDEL *N. benthamiana* plants did not show any green fluorescence in the chloroplast envelope (Figure 3I) and western blots were negative for the presence of HDEL (a marker for the ER; Figure 3J), confirming that the TGB2 protein (Figure 3F) was indeed associated with the chloroplast envelope and not contaminating ER membranes.

### Detection of PMTV negative strand RNA in chloroplasts

In order to determine whether viral replication was occurring at chloroplasts, RT-PCR was used to detect the viral replicative intermediate, negative strand RNA in chloroplasts isolated from PMTV-Swe-infected plants. Non-infected *N. benthamiana* leaves were used as a negative control. No negative strand RNA was detected in healthy control chloroplasts, but was detected in each sample of PMTV-infected chloroplasts (Figure 3K) indicating that replication of viral RNA occurs in association with chloroplasts.

### *In-situ* viral RNA localisation

**Figure 4:**
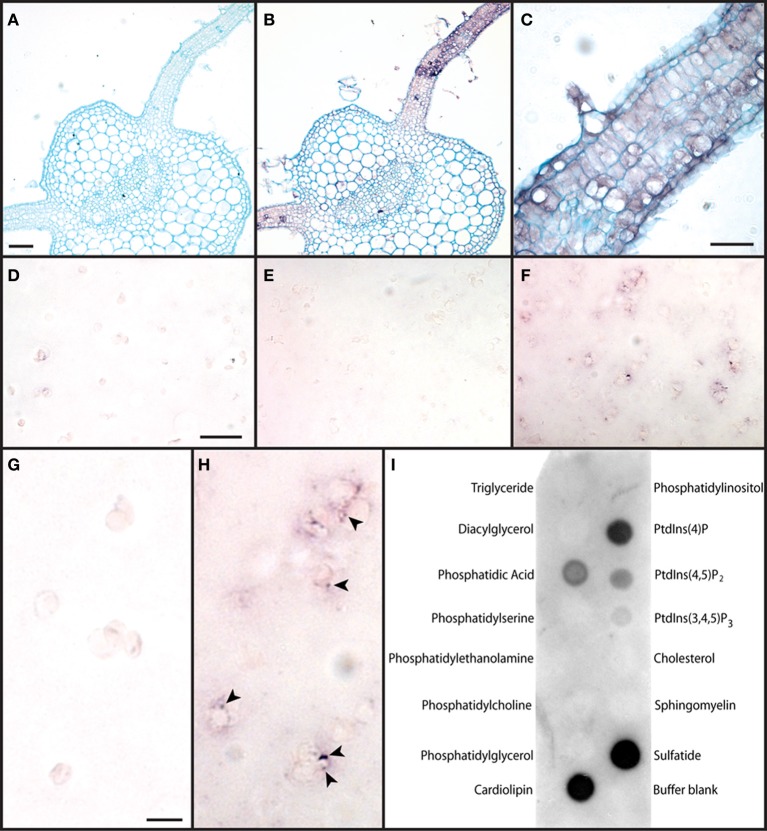
**Association of PMTV RNA with chloroplasts and TGB2 with lipids. (A)** PMTV 3′UTR DIG-labeled sense RNA probe did not detect any viral RNA in a section of leaf infected with PMTV as shown by the lack of purple precipitate. The tissue has been counterstained with Alcian Blue to show cell structure. (Bar = 250 μm for **A** and **B**). **(B)** PMTV 3′UTR DIG-labeled antisense RNA probe detected viral RNA in the leaf lamina and in some parenchyma cells associated with the vascular trace in the midrib of the leaf, as shown by purple-coloration of infected cells with replicating virus. The tissue has been counterstained with Alcian Blue to show cell structure. **(C)** A higher magnification image of the tissue shown in **(B)** to show the purple coloration associated with viral RNA in many cell types in a patch of PMTV-infected leaf lamina (Bar = 100 μm). **(D)** Chloroplasts from control, healthy tissue did not label strongly with the PMTV DIG-labeled antisense probe showing that there was little non-specific labeling with this probe (Bar in D = 20 μm for **D–F**). **(E)** Chloroplasts from PMTV-infected tissue did not label strongly with the PMTV DIG-labeled sense probe showing that, if present, the viral negative strand RNA which is produced as a replicative intermediate was present at levels too low to be detected. **(F)** Chloroplasts from PMTV-infected tissue and labeled with the PMTV DIG-labeled antisense probe showed viral genomic RNA associated with the plastid membrane. Small purple spots can be seen in many chloroplasts in this image. **(G,H)** Show higher-magnification images of portions of **(D)** and **(F)** respectively. Some of the spots of viral RNA associated with the plastid membrane are marked with arrowheads in **(H)**. (Bar in G = 5 μm for **G** and **H**). **(I)** Membrane blot showing TGB2 interactions with a number of lipids. The strongest reactions were obtained with cardiolipin and sulfatide, but PtdIns(4)P, Phosphatidic acid, PtdIns(4,5)P_2_ and PtdIns(3,4,5)P_3_ also showed interactions of decreasing intensity with TGB2.

To further confirm the presence of viral RNA at chloroplasts, *in-situ* RNA labeling of PMTV-Swe-infected *N. benthamiana* leaves and isolated chloroplast preparations was conducted. Antisense and sense DIG-labeled RNA probes, designed to hybridize to and detect viral genomic RNA and the replicative intermediate respectively, were first tested on healthy and PMTV-infected leaf tissue. No viral RNA was detected using either probe in healthy tissue (data not shown). The sense probe did not detect any replicative intermediate (negative strand) RNA in sections of PMTV-infected leaf midrib and lamina (Figure 4A), but on adjacent serial sections, the antisense probe showed the presence of viral RNA in all infected cells. PMTV was found in a patchy manner throughout the tissue, but especially in the leaf lamina (Figures 4B and C), and although not high-resolution images, these micrographs suggested the probe was associated with chloroplasts.

Viral genomic RNA was also detected in association with isolated chloroplasts; eighty random fields of labeled chloroplasts were selected and between 2 and 15% of plastids in each field showed the presence of positive-strand RNA. The PMTV antisense probe did not react strongly with chloroplasts from healthy tissue (Figures 4D and G), nor did the sense probe with chloroplasts from PMTV-infected tissue (Figure 4E), but viral genomic RNA was associated with chloroplasts from PMTV-infected tissue as detected by the antisense probe (Figures 4F and H). In these chloroplasts the RNA appeared to be in punctate spots associated with the periphery of the chloroplasts (arrows in Figure 4H).

### Protein-lipid assays

Since mRFP-TGB2 interacts with a number of different sub-cellular compartments we wanted to ascertain the specificity of membrane targeting. Membrane lipid arrays were incubated with preparations of recombinant thioredoxin-TGB2 fusion protein, which was subsequently detected with antiserum raised to the fusion protein. The results showed that TGB2 interacted with 6 of the 15 lipids tested: cardiolipin, 3-sulfogalactosylceramide (sulfatide), phosphatidic acid and the phosphoinositides PtdIns(4)P, PtdIns(4,5)P_2_, and PtdIns(3,4,5)P_3_. Reproducible results were obtained using TGB2 at either 20 μgml^−1^ or 2 μgml^−1^, and a representative assay is shown in Figure 4I. No reactions were seen in controls where the arrays were incubated with thioredoxin followed by the thioredoxin-TGB2 antiserum and anti-rabbit HRP, showing the binding was TGB2-specific (data not shown).

### Electron microscopy of infected plants reveals the presence of abnormal cytoplasmic inclusions in chloroplasts

**Figure 5:**
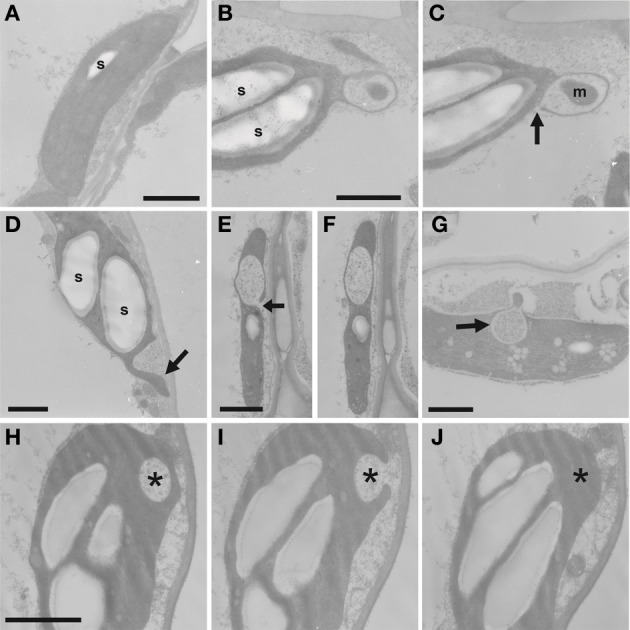
**Ultrastructural effects of PMTV infection on chloroplasts.** Transmission electron microscope images of thin sections from a healthy N. *benthamiana* plant **(A)**, PMTV-infected *N. benthamiana* plants **(B–F)** and *S. tuberosum* cv. Scarborough **(G–J)**, the sections show ultrastructural changes in chloroplasts. **(A)** Example of a typical chloroplast from an uninfected *N. benthamiana* plant containing a single small starch grain (s). Bar = 1 μm. **(B,C)** Sequential sections through a distorted chloroplast, from a PMTV-infected plant, containing large starch grains (s) and with a terminal, cytoplasmic inclusion encompassing a mitochondrion (m): in (**B**) the inclusion appears enclosed, while in **(C)** it appears open with the aperture to the cytoplasm marked with an arrow. Bar in B = 1 μm for (**B** and **C**). **(D**) Chloroplast containing large starch grains (s) and with an abnormal, terminal extension (marked with arrow). Bar = 1 μm **(E,F**) Serial sections through a chloroplast of an epidermal cell with a cytoplasmic inclusion: the inclusion appears open in **(E)** (aperture marked with an arrow) and enclosed in **(F)**. Bar in E = 1 μm for **(E** and **F). (G)** Chloroplast with large cytoplasmic inclusion (marked with an arrow) from PMTV-infected potato plant. Bar = 1 μm. **(H,I,J)** Serial sections through a chloroplast from a potato plant infected with PMTV: in **(H)**, the inclusion (*) appears enclosed, while it is open in **(I)** and missing from **(J)**, indicating the open flask-shaped nature of these inclusions. (Bar in H = 1 μm for **H–J**).

Previous electron microscopical reports of PMTV infection have not shown detailed cytological effects of the virus and so ultra-structural studies were used to investigate the physical effects of virus infection on chloroplasts. Tissue samples from leaves of healthy or virus-infected *N. benthamiana* plants were fixed and embedded, and thin sections were examined in the electron microscope. PMTV infections in leaves typically have a low titre of virions and viral spread is sparse and patchy within leaves; tissue was sampled from uninfected leaves and infected leaves showing yellowing symptoms. The most marked difference between the samples was the presence of multiple large starch grains in chloroplasts in infected leaves, while chloroplasts in healthy leaves had none or a few small starch grains (Figures 5A and B). The chloroplasts showed extensive deformation and often had large cytoplasmic inclusions (Figures 5B–D) and abnormal terminal projections (Figure 5D). These inclusions occurred most frequently at the ends of chloroplasts and often encompassed organelles such as mitochondria (Figures 5B and C). The frequency of chloroplasts with inclusions and terminal projections was more prevalent in older infected tissue with yellow mosaic symptoms than in younger infected tissue. Such abnormalities were not found in uninfected tissue. The form of the cytoplasmic inclusions was assessed by examination of serial ultra-thin sections. They were found to be spheroidal in shape, with diameters of about 0.5–1 μm, but were also not true inclusions; rather they were flask-shaped, cytoplasmic invaginations, each being connected to the external cytoplasm by a narrow opening (Figures 5C,E,F). The incidence of virus particles in infected tissue was very low and particles were not observed in the flask-shaped invaginations.

Ultra-structural studies were also carried out on leaf tissue from the natural host, potato. As in the experimental host, the most pronounced abnormality was the presence of multiple, large starch grains in chloroplasts and some contained cytoplasmic inclusions (Figure 5G), which were most frequently found at the ends of chloroplasts. Examination of serial sections from infected potato tissue showed that these inclusions were also spheroidal and connected to the ground cytoplasm by a cytoplasmic bridge (Figures 5H–J).

## Discussion

It was previously shown that PMTV mRFP-TGB2 and GFP-TGB3 when expressed from a 35S promoter co-localized in cellular membranes and mobile granules, utilizing the ER-actin network to facilitate movement to the cell periphery and PD. In addition, TGB2 and TGB3 associated with components of the endocytic pathway. The SEL was also increased in cells expressing either protein, suggesting that both proteins can interact with, and functionally gate, PD (Haupt et al., 2005). In this study the distribution of mRFP-TGB2 was examined after expression from a modified PMTV vector under the control of a viral subgenomic promoter to investigate whether the observed localizations were reproducible in a virus context. Identical results were obtained with the different expression systems used and a more detailed analysis of the images of the ~4 μm-diameter spherical structures first published in Haupt et al. (2005) where they were simply referred to as large vesicles, revealed that they were chloroplasts. This finding was surprising because chloroplasts are typically ovoid or disc-shaped organelles (Esau, 1965), but this rounding-up of chloroplasts has also been seen before in BSMV-infected tissues (Torrance et al., 2006). Movement of a PMTV mutant clone that was deficient in TGB2 expression was functionally complemented by mRFP-TGB2, allowing the virus, as reported by YFP-TGB1 fluorescence, to move into neighboring cells. Following co-expression, YFP fluorescence was detected in cells surrounding the initial infected cell and occasionally more than one cell boundary distant. In these experiments, the proportion of co-expressing cells was low in comparison to the numbers of individual red- or yellow-fluorescent cells. This may be a result of exclusion of the virus due to a form of RNA silencing, since the PMTV-3.YFP-TGB1.ΔTGB2 mutant was created by mutation of the TGB2 initiation codon rather than by deletion of the gene. It is highly unlikely that cell-to-cell movement of the TGB2 deletion mutant was due to reversion since, in control bombardments of RNA1 and the mutant viral RNA, movement out of the initial cell never occurred. Furthermore, if the mutant had reverted, continued movement over many cell boundaries, resulting in larger fluorescent lesions of greater than 20–100 cells, would have been expected. The bombardment results confirm that TGB2 is not required for viral replication, although PMTV TGB2 is known to bind RNA (Cowan et al., 2002).

These data reveal subtle differences in the localizations of PMTV TGB2 protein compared to other TGB2 proteins. In the systems examined, TGB2 proteins are all observed in association with the ER and mobile granules, particularly at early time points in expression. However, unlike PMTV, the BSMV TGB2 localized later in the infection to chloroplasts only in the presence of viral RNA (Torrance et al., 2006) and PVX TGB2 has never been reported to associate with chloroplasts or with components of the endocytic pathway, accumulated instead in the cytosol and nucleus later in expression (Ju et al., 2005).

Chloroplasts labeled with mRFP-TGB2 were often seen surrounding the nucleus and mRFP-TGB2 also labeled stromules. In the experiments reported here, localization to the chloroplast envelope was consistent between different expression systems, although the timing varied slightly. The localizations described for TGB2 from a PMTV vector under the control of its own subgenomic promoter were all visible slightly later than from 35S promoter expression. Differences in the time at which localizations were observed may be due to different levels of expression between the various systems rather than an intrinsic effect of the expression system, however, we cannot rule out the possibility of temporal regulation by the virus infection process. Deconvolution has produced greater resolution of fine and faintly-labeled structures, showing additional detail and allowing imaging of compartments that were previously invisible without image processing. It was, for instance, interesting to note that, when deconvolved, the ER and small mobile structures were still visible at the later stage of chloroplast labeling. These sub-cellular structures are typically labeled with mRFP-TGB2 early in the infection process (see Haupt et al., 2005), but were previously considered to be unlabeled at the later, vesicle and chloroplast-associated stage.

TGB2 was undetectable in preparations of chloroplasts from PMTV-infected tissues using antisera raised against TGB2, and in a previous study we were unable to detect TGB2 in PMTV systemically-infected *N. benthamiana* tissue. However, TGB2 was detected in P1 and P30 fractions (enriched for nuclei, chloroplasts and cellular membranes) when it was over-expressed from a TMV-based vector (Cowan et al., 2002). In the present study, TGB2 was found, by CLSM imaging, to be present in ~5% of the intact chloroplasts isolated from tissues that were systemically infected with TMV expressing GFP-TGB2. It seems likely therefore, that the level of expression of TGB2 in natural infections is below the limit of detection in Western blots. However, the distribution of mRFP-TGB2 fluorescence in chloroplasts was clear in the CLSM experiments reported here using three different expression systems. These results suggest that, in a natural infection, TGB2 is present on and in chloroplasts and that this localization is a functional part of the infection process, but that the levels required for this function are below the detection limits of the available antiserum. The technical difficulty in preparing chloroplasts for analysis is compounded by the presence of large starch grains in chloroplasts from PMTV-infected tissues (cf Figure 4): these tend to pass through the plastid membrane during centrifugation, rupturing the chloroplast and probably reducing the abundance of chloroplasts from infected cells in any preparation used for analysis.

RT-PCR detected the presence of viral negative strand RNA (the replicative intermediate), in chloroplasts isolated from PMTV-Swe-infected *N. benthamiana* leaves, indicating that replication of viral RNA occurs in association with chloroplasts. *In-situ* RNA labeling of both infected tissue and isolated chloroplasts from infected tissue showed that viral genomic (positive strand) RNA was associated with infected plastids. *In-situ* RNA labeling was unable to detect negative strand RNA above background labeling, but this is perhaps not surprising since it is assumed to be present in very small quantities; methods utilizing PCR amplification are likely to be of greatest utility. Detection of viral RNA in association with chloroplasts was also a relatively rare event; only a small percentage of isolated chloroplasts or small patches of leaf tissue showed hybridization with the PMTV probe which is consistent with the sporadic and patchy nature of a PMTV infection in foliar tissue. When detected, the viral genomic RNA was found to be in punctate spots associated with the plastid periphery; possibly corresponding to the membrane invaginations seen in the EM and the punctate spots of TGB2 seen with CLSM.

Because TGB2 was localized to several different subcellular compartments we wanted to investigate whether TGB2 displayed any binding specificity to membrane lipids. Lipids play important roles in the maintenance and activity of integral membrane proteins and function as signaling molecules in plant growth and development (reviewed by Martin et al., 2005), and can be involved in plant pathogen resistance (Takahashi et al., 2009) and viral replication (Lee and Ahlquist, 2003). Lipid interaction analysis revealed that TGB2 interacts with phosphatidic acid, a signaling phospholipid and biosynthetic precursor of cardiolipin. Phosphatidic acid is thought to be transported from the ER to chloroplast thylakoids (Awai et al., 2006), and cardiolipin is present in photosystem II fractions from chloroplast thylakoid membranes (Depalo et al., 2004), both providing additional supporting evidence for TGB2 association with plastids. The other lipids that TGB2 interacted with in the membrane blots, the phosphoinositides (PtdIns) and sulfatide are found in discrete domains of plasma membranes and are involved in signaling and host-pathogen interactions (Mongrand et al., 2004; Borner et al., 2005). These interactions are the subject of further study. The lipid binding experiments support the contention that TGB2 interacts with specific lipid domains since no reaction was found with phosphatidylcholine and phosphatidylethanolamine both of which are major constituents of plasma membranes.

There have been many reports of viruses inducing deformation and disruption of chloroplasts. For example, chloroplasts are known to be sites of replication of TYMV where the virus induces small spherules at the periphery (Prod'homme et al., 2001). The 6K protein of (TuMV) induces vesicles that target chloroplasts and induce chloroplast membrane invaginations (Wei et al., 2010) and virus infection causes plastids to accumulate in perinuclear structures containing other membraneous organelles and viral proteins that are required for genome replication (Grangeon et al., 2012). BSMV induces cytoplasmic invaginations and vesiculation in membranes of proplastids and chloroplasts (Carroll, 1970; Torrance et al., 2006). The CP and RNA of BSMV were detected in chloroplast preparations (Torrance et al., 2006) while double-stranded RNA was found in proplastids of wheat root tips (Lin and Langenberg, 1985), implicating plastids as sites of virus replication. AltMV TGB3 targets to chloroplasts, causes chloroplast malformation and vesicular membrane invaginations and the association is required for efficient cell-cell and systemic movement. As found for PMTV, viral RNA also associates with the chloroplast periphery (Lim et al., 2010). Viruses commonly cause chlorosis and have been reported to adversely affect photosystem II structure and function (Rahoutei et al., 2000; Lehto et al., 2003). Recently, it was shown that the potyvirus helper component proteinase (HC-Pro), a multifunctional protein with roles in RNA silencing suppression, movement and transmission by vector aphids, interacts with the tobacco MinD protein that plays a role in chloroplast division (Jin et al., 2007). It was suggested that HC-Pro binding may interfere with chloroplast division to promote virus pathogenicity.

A previous EM study of PMTV-infected potato tissue revealed an abundance of cytoplasmic membranous tubules and perforation and loss of both the plasma membrane and chloroplast envelope (Fraser, 1976). However, chloroplast ultra-structure was not reported in detail. In our study, we show for the first time that PMTV induces the formation of large cytoplasmic invaginations in chloroplasts and tests on chloroplast preparations from PMTV-infected *N. benthamiana* showed that viral genomic RNA and CP were associated with chloroplasts. Immunogold labeling of ultrathin sections with the anti-TGB2 antibody was unable to detect TGB2 protein in association with the chloroplasts, but this is perhaps unsurprising since the TGB2 antiserum did not work in western blots. However results presented here show TGB2 associated with plastid membranes and localized to spots on the surface of chloroplasts (possibly cytoplasmic invaginations) using a number of different methods. The additional association of viral RNA and CP with the spots on chloroplasts suggests these may be sites of virus replication, and it is possible that the plastid association of TGB2 reflects a role in viral replication by recruiting vRNP. Alternatively it is possible that TGB2 has a cytopathic effect, for example, blocking or subverting chloroplast receptors thereby inducing inclusions of cytoplasm for replication. A similar phenotype (cytoplasmic inclusions) has been observed in mutants defective in chloroplast import receptors (Kubis et al., 2004). Chloroplasts are sites of salicylic acid biosynthesis and are the target of virulence effectors in other host-pathogen interactions (Jelenska et al., 2007). Therefore, it is possible that chloroplast targeting by TGB2 is virus-mediated to promote pathogenesis. However, we do not know whether PMTV induces a salicylic acid mediated defense response. It may also be that TGB2 is required for intracellular transport of viral RNP complexes or assembled virions that are produced in association with plastids; TGB2 associates with TGB3 in a complex of defined stoichiometric ratio to associate with motile membrane compartments of the ER through protein-protein or protein-lipid interactions to achieve passage to and through PD (Tilsner et al., 2010). Although virus particles were not seen associated with the invaginations of chloroplasts in EM sections in this study, CP was detected in chloroplast preparations, suggesting that CP production could be associated with plastid membranes, or encapsidation could occur here. PMTV does not produce large quantities of virions in infected cells, but given that the virus can move as a vRNP complex, virions may not be important in leaf tissue. The one event that does require the viral CP is natural transmission by its plasmodiophorid vector, *Spongospora subterranea*, but this only occurs in root cells. This lack of necessity for virions to move systemically through the apical parts of the plant may explain their relative absence in infected leaf cells. Future research will be focused on establishing whether TGB2 plays a role in pathogenesis, vRNP and virion transmission and/or virus replication.

### Conflict of interest statement

The authors declare that the research was conducted in the absence of any commercial or financial relationships that could be construed as a potential conflict of interest.
